# M2 Receptor Activation Counteracts the Glioblastoma Cancer Stem Cell Response to Hypoxia Condition

**DOI:** 10.3390/ijms21051700

**Published:** 2020-03-02

**Authors:** Ilaria Cristofaro, Chiara Limongi, Paola Piscopo, Alessio Crestini, Claudia Guerriero, Mario Fiore, Luciano Conti, Annamaria Confaloni, Ada Maria Tata

**Affiliations:** 1 Department of Biology and Biotechnologies Charles Darwin, Sapienza, University of Rome, 00185 Rome, Italy; ilaria.cristofaro@uniroma1.it (I.C.); chiara.limongi@uniroma1.it (C.L.);; 2 Depatment of Neuroscience, Istituto Superiore di Sanità, 00161 Rome, Italy; paola.piscopo@iss.it (P.P.); alessio.cristini@iss.it (A.C.); 3 IBPM, Institute of Molecular Biology and Pathology, CNR, 00185 Rome, Italy; mario.fiore@uniroma1.it; 4 Department of Cellular, Computational and Integrative Biology—CIBIO, University of Trento, 38123 Trento, Italy; luciano.conti@unitn.it; 5 Research center of Neuroscience, Sapienza, University of Rome, 00185 Rome, Italy

**Keywords:** cancer stem cells, glioblastoma, hypoxia, M2 muscarinic receptor, miR-210

## Abstract

Glioblastoma multiforme (GBM) is the most malignant brain tumor. Hypoxic condition is a predominant feature of the GBM contributing to tumor growth and resistance to conventional therapies. Hence, the identification of drugs able to impair GBM malignancy and aggressiveness is considered of great clinical relevance. Previously, we demonstrated that the activation of M2 muscarinic receptors, through the agonist arecaidine propargyl ester (Ape), arrests cell proliferation in GBM cancer stem cells (GSCs). In the present work, we have characterized the response of GSCs to hypoxic condition showing an upregulation of hypoxia-inducible factors and factors involved in the regulation of GSCs survival and proliferation. Ape treatment in hypoxic conditions is however able to inhibit cell cycle progression, causing a significant increase of aberrant mitosis with consequent decreased cell survival. Additionally, qRT-PCR analysis suggest that Ape downregulates the expression of stemness markers and miR-210 levels, one of the main regulators of the responses to hypoxic condition in different tumor types. Our data demonstrate that Ape impairs the GSCs proliferation and survival also in hypoxic condition, negatively modulating the adaptive response of GSCs to hypoxia.

## 1. Introduction

Glioblastoma multiforme (GBM) is the most common and aggressive form of primary brain tumor. Individuals affected by this tumor have a prognosis of 12–15 months of life after standard treatment. Only the 3–5% of patients survive up to 5 years after diagnosis [1]. GBM develops mainly in the cerebral hemispheres or sub-territorially in the cerebral trunk and in the cerebellum [2].

Local invasiveness, neo-angiogenesis and intra-tumoral heterogeneity are the main hallmarks of GBM. The challenges in the treatment of GBM are strongly related to the site where the tumor develops and its complex and heterogeneous biology [3]. Advances in surgical, radiotherapy and chemotherapy approaches have allowed a gradual improvement in the survival and quality of life of GBM patients, but the prognosis is unfortunately still not very encouraging. Surgery is the standard treatment, where possible. Surgical treatment can be followed by radiotherapy and chemotherapy to eradicate residual tumor cells [4,5]. Given the intrinsic genetic and phenotypic heterogeneity of GBM, it is possible that current standard treatments eliminate only specific and more susceptible GBM subpopulations, while the more resistant ones survive and repopulate the tumor. This leads to a more aggressive recurrent tumor that does not respond to initial therapy and significantly compromises the patient’s prognosis.

The discovery of GBM stem cells (GSCs) as undifferentiated cancer cell subpopulation helps to explain the aggressive phenotype, the possibility of recurrence and resistance to GBM treatments [6]. The high DNA repair capability of GSCs increases resistance to apoptosis and the drug efflux system are among the cellular mechanisms used by GSCs to elude chemotherapy and radiotherapy [7].

Hypoxia is defined as a state of low availability of O_2_ that limits or even abolishes the functions of organs, tissues and cells. This state occurs in a wide variety of pathological conditions such as tissue ischemia, inflammation and tumors.

In particular in the solid tumors, the O_2_ supply to neoplastic cells is often reduced or even abolished. The process of tumor progression is characterized by rapid cell growth and by changes in the microenvironment due in large part to an insufficient supply of O_2_. Hypoxia affects many aspects of the tumor’s biology and their response to therapy [8].

One of the main cellular events that occurs following the exposure to hypoxia is the activation of Hypoxia-inducible-factor (HIF-1). Under conditions of reduced O_2_ concentrations, HIF-1α (the subunit of HIF heterodimer oxygen-sensitive) binds to the elements responsive to hypoxia (HREs) and induces the transcription of various target genes involved in tumor angiogenesis, glucose metabolism, invasiveness and cell survival [9]. In particular, the induction of HIF-1α allows the expression of genes that promote the reprogramming of tumor metabolism towards the glycolic pathway, increasing glucose uptake, the expression of glycolic enzymes and the production of lactate, regulating the pyruvate metabolism. In order to promote tumor growth and survival, HIF-1α activation increases the expression of many pro-angiogenic factors, including vascular endothelial growth factor (VEGF), Vascular Endothelial Growth Factor Receptor (VEGFR), angiopoietins (ANG-1 and ANG-2), and metalloproteinases such as MMP-2 and MMP-9, which support the vascular remodeling of the tumor invasiveness [10]. 

GSCs are present both in vascular and necrotic/hypoxic niches. These niches are not only the anatomical and structural units in which the stem cells reside, but they represent functional and specialized microenvironments that support proliferation, nutrients diffusion and regulate the capacity of self-renewal and cell fate decision [7,11].

Hypoxia plays a key role in maintaining the stem cell phenotype of GSCs by activating molecular players such as Sox2, Oct4, Nanog and Notch-1. Moreover, it strongly influences the therapeutic resistance of cancer cells, because on one hand it has a negative impact on radiation treatment (radiation requires O_2_ to have a maximum cytotoxic effect), on the other hand, it hinders the effectiveness of some drugs, upregulating the expression of O^6^-methylguanine DNA methyltransferase (MGMT) and ATP-binding cassette (ABC) transporters [12]. In synthesis, hypoxia promotes survival and increases the aggressiveness of cancer stem cells. 

Acetylcholine muscarinic receptors are widely distributed both in the central and peripheral nervous system, and in several mammalian organs [13]. Several in vitro and in vivo studies indicate that the activation of M3 receptors enhanced tumor cell proliferation [14,15]. Conversely, our previous studies have shown that M2 receptors activation by arecaidine propargyl ester (Ape) is able to arrest cell proliferation in GBM cell lines (U87MG and U251MG) and GSCs (GB7 and GB8 cells) [16,17,18]. Moreover, M2 receptor activation induces oxidative stress and severe apoptosis, significantly reducing cell survival in particular in GBM-p53 mutated [19]. 

Based on data previously obtained, in the present study, we evaluated the effects mediated by M2 muscarinic receptor activation on the adaptation of GSCs to hypoxic stress with the aim of assessing whether the M2-mediated action can counteract hypoxic responses by interfering with the mechanisms that regulate the acquisition of a more aggressive tumor phenotype. 

The results obtained demonstrated that in hypoxia, the M2 receptors activation caused the inhibition of GSC cell cycle progression, causing a significant increase of aberrant mitosis with consequent decreased cell survival. qRT-PCR has also indicated that Ape downregulates the expression of stemness marker CD133 and miR-210 expression, one of main regulators of hypoxia adaptive responses in different tumors.

## 2. Results

### 2.1. M2 Receptor Activation Negatively Modulates GSCs Adaptation to Hypoxia Condition

GB7 cells were plated on a laminin-coated plastic and then placed in normoxic or hypoxic conditions.

**Figure 1:**
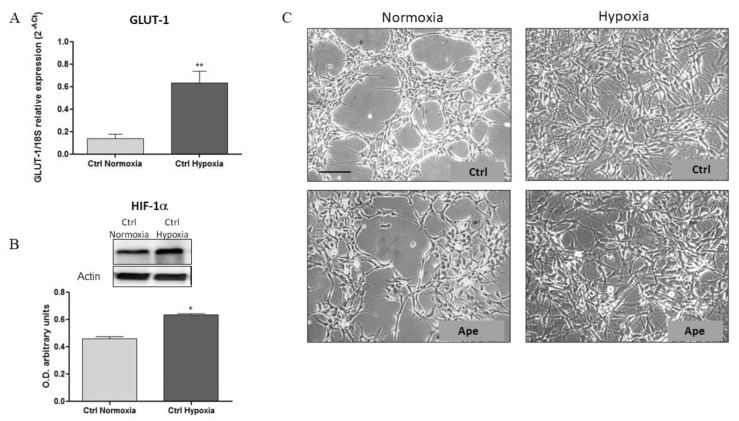
(**A**) Real-Time PCR analysis of GLUT-1 transcript expression, in GB7 cells in normoxia and after 24 h hypoxia. The data are the mean ± SEM of the three independent experiments performed in duplicate (*t*-test: ** *p* < 0.01). (**B**) Western blotting analysis of the Hypoxia-inducible-factor (HIF-1α) protein in GB7 cells in normoxia and 24 h hypoxia. The graph below shows the densitometric analysis of the bands obtained after normalization with the β-actin as housekeeping reference protein (*t*-test; * *p* <0.05). The data are the mean ± SEM of the three independent experiments. (**C**). GB7 cells in normoxia and hypoxia. After 24 h of 100 μM arecaidine propargyl ester (Ape) treatment the cells present a more dispersed organization, more evident in hypoxia (scale bar = 150 µm).

To validate the hypoxic condition, we evaluated the expression of markers that are generally upregulated in a state of oxygen deprivation, including GLUT-1 and HIF-1α. Transcriptional analysis showed a significant up regulation of GLUT-1 transcript at 24 h of exposure to the hypoxic condition (Figure 1A). Similarly, we found a significant increase in HIF-1α protein levels in the hypoxic samples when compared with the normoxic one (Figure 1B)

**Figure 2:**
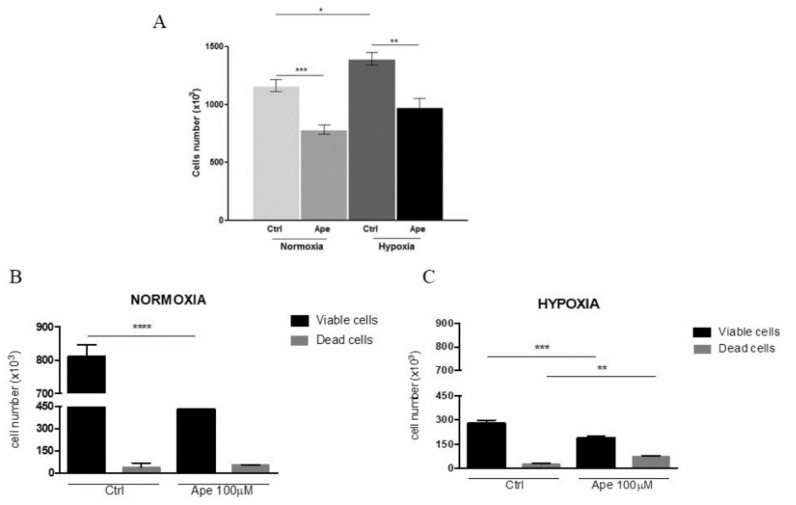
(**A**) Cell number was measured in normoxia and hypoxia and in presence or absence of 100 μM Ape (24 h). (**B**) Viable and dead cells (revealed by trypan blue staining) were measured after 48 h in normoxia and in presence or absence of 100 μM Ape; (**C**) Viable and dead cells were measured after 48 h in hypoxia and in presence or absence of 100 μM Ape.

After 24 h of exposure to the hypoxic condition, GSCs lost their typical spatial arrangement and spread on all the space available (Figure 1C), showing a significant increase of cell number (Figure 2A). As previously reported, the treatment with M2 agonist Ape (100 μM) causes a significant decrease of cell number [18]. Interestingly, M2 agonist activation also caused a decrease of cell number in hypoxic condition (Figure 2A). Moreover, a further reduction in cell number was evident after 48 h of Ape treatment both in normoxia and hypoxia, accompanied by an initial increase in the number of dead cells but only in hypoxia condition (Figure 2B,C). Remarkably, after 48 h of hypoxia, a significant reduction of cell number was also evident in absence of Ape treatment (Figure 2B,C). 

**Figure 3:**
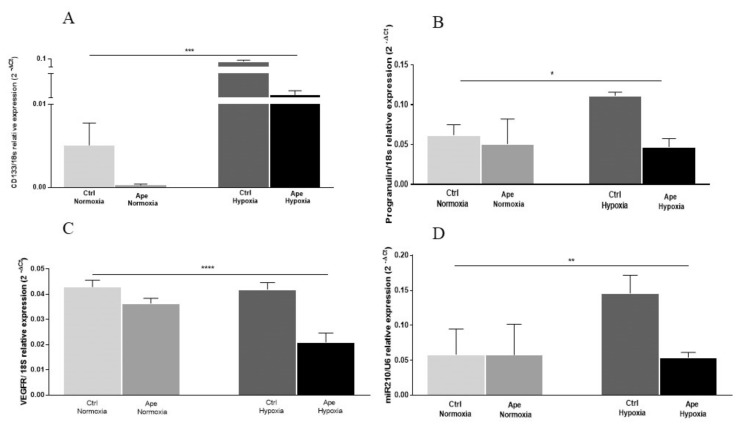
qRT-PCR analysis of CD133 (**A**), progranulin (PRGN) (**B**), VEGFR (**C**) and miR-210 (**D**) transcripts levels, in the GB7 cell line in normoxia and after 24 h hypoxia and in presence or absence of 100 μM Ape (24 h). The data are the mean ± SEM of the three independent experiments performed in triplicate. (ANOVA test; * *p* <0.05: ** *p* < 0.01; *** *p* < 0.001; **** *p* < 0.0001)**.**

The increase of cell number observed after 24 h of hypoxia (Figure 2A) seems to be associated with a significant increase of CD133 transcript levels (Figure 3A). CD133, also known as Prominin-1, is one of the common stemness marker for GSCs. As expected, hypoxic stress induces a significant upregulation of the expression of CD133, suggesting that hypoxia promotes the stemness property in GSCs (Figure 3A). Treatment with Ape significant decreases CD133 mRNA levels both in the normoxic and hypoxic condition, suggesting that the activation of the muscarinic M2 receptor is able to counteract the stemness property in GB7 cells (Figure 3A). 

In order to evaluate whether the treatment with Ape was able to counteract the cellular response to hypoxic stress, we evaluated by Real-Time PCR the expression of genes induced by the hypoxic microenvironment, linked to an increase of tumor aggressiveness.

Progranulin (*PGRN*) is a protein involved in tumorigenesis; it promotes cell proliferation and invasiveness and its expression is upregulated under hypoxic conditions [20,21]. We evaluated the expression levels of the *PGRN* transcript in the GB7 cells in different experimental conditions. By qRT-PCR analysis, we found a significant upregulation of *PGRN* mRNA levels following 24 h of exposure to O_2_ deprivation (Figure 3B). Treatment with Ape significantly counteracts the expression of the *PGRN* transcript, but only in hypoxic conditions (Figure 3B). Moreover, we evaluated the expression of the vascular endothelial growth factor receptor, VEGFR. This receptor mediates the response to the VEGF stimulation, promoting neo-angiogenesis. Treatment with M2 agonist induced a downregulation of VEGFR receptor expression both in normoxia and hypoxia (Figure 3C). 

MiR-210, the master hypoxamir, is a main factor regulating HIF, whose expression levels may be a reflection of the activity of this transcription factor [22]. miR-210 is a nodal molecular component linked to the microenvironment, cellular metabolism and the clinical course of tumor pathology [23]. As expected, this miRNA was significantly upregulated in GB7 cells after exposure to hypoxia (Figure 3D). Treatment with M2 agonist Ape induced a significant reduction of miR-210 expression, but only in hypoxia (Figure 3D). 

### 2.2. M2 Receptor Activation Arrests Cell Cycle Progression and Induces Aberrant Mitosis

**Figure 4:**
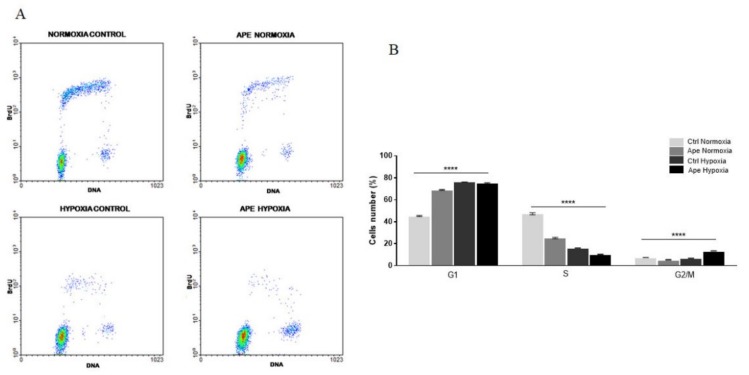
(**A**) Bi-parametric analysis of BrdU incorporation and DNA content in GB7 cells in normoxia and hypoxia and in presence or absence of 100 μM Ape (24 h); (**B**) Percentage of GB7 cells in G1, S, G2 /M phases in the different experimental conditions. The data are the mean ± SEM of the three independent experiments (ANOVA test; **** *p* <0.0001).

Considering the decreased cell growth previously described, we further investigated the cell cycle progression in hypoxic condition and upon Ape treatment. By flow cytometry (FACS) analysis, after BrdU incorporation, we observed a significant reduction of the cell percentage in S phase following Ape treatment both in normoxia and hypoxia (Figure 4A,B). In the hypoxic control sample, a different distribution of the cell population is evident if compared to the corresponding control sample in normoxia. In fact, the Ape treatment caused an increase of cell percentage in G1 phase and corresponding reduction of cells in S phase in normoxic condition. Conversely, in hypoxia, we observed that Ape treatment caused a decrease of cells in S phase, with an accumulation in G2/M phase, while the percentage of cells in G1 phase remained substantially untouched (Figure 4B).

**Figure 5:**
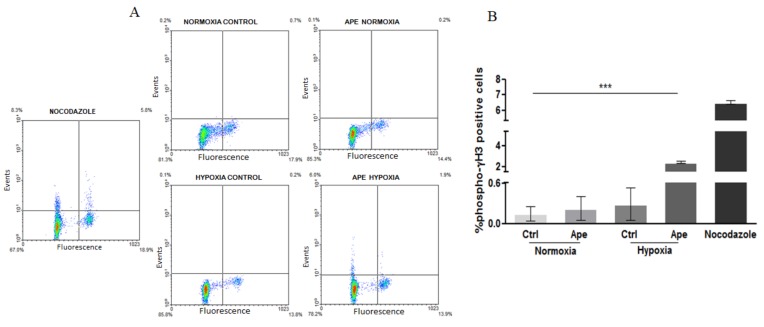
(**A**) (FACS) analysis of the cell positive for p-H3 in normoxia and hypoxia and in presence or absence of 100 μM Ape (24 h). Nocodazole (0.2 μg/mL) was used as positive control. (**B**) Percentage of phospho-Histone H3 positive cells in different experimental conditions. The data are the mean ± SEM of the three independent experiments (*t*-test analysis *** *p* <0.001; Ape hypoxia *vs* all experimental conditions).

In order to discriminate between a block in phase G2 or in M phase, we evaluated the percentage of cells positive for histone 3 phosphorylated in serine 10 (pH3), a highly condensed chromatin marker specifically expressed during M phase. The treatment with 100 μM Ape (24 h) in hypoxic condition caused an increase of the pH3-positive cells (Figure 5 A,B) only in hypoxia. In fact, in normoxia, the percentage of cells in M phase both in control and Ape-treated sample was comparable.

**Figure 6:**
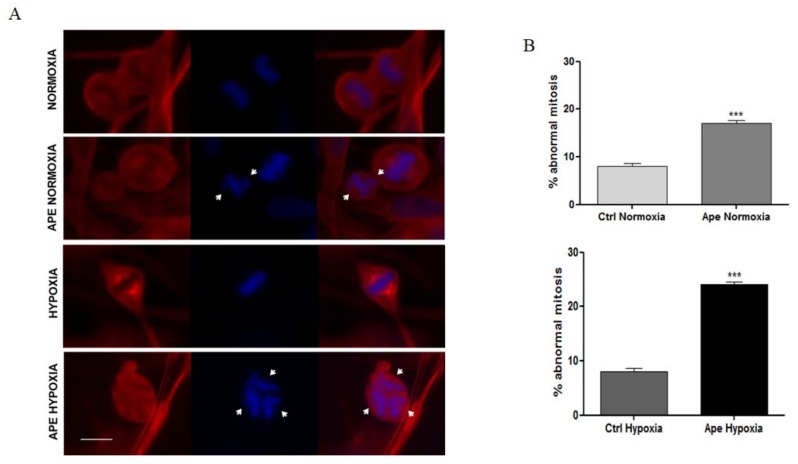
(**A**) Immunocytochemistry of the GB7 cells for α-tubulin (Red) in normoxia and hypoxia and in presence or absence of 100 μM Ape (24 h). (DAPI) (Blue) was used for DNA and chromosome staining (scale bar = 5 µm) (**B**) Percentage of abnormal mitosis measured in three independent experiments (*t*-test; *** *p* < 0.001).

By immunocytochemistry, we analyzed the cell division in GB7 cells by α-tubulin and 4′,6-diamidino-2-phenylindole (DAPI) labelling to mark the mitotic spindles and chromosomes, respectively. The evaluation of the cells presenting metaphases with chromosomes, aligned or misaligned, allowed to identify the presence of abnormal mitosis. Both in normoxia and hypoxia, the untreated groups (control) presented about 8% of cells showing abnormal mitotic divisions (Figure 6A,B). Conversely, Ape-treated cells in normoxia showed a significant increase of the percentage of abnormal mitosis (17%) (Figure 6A,B). In particular, a greater number of abnormal mitosis was found in Ape-treated GSCs in hypoxic condition (24%). Moreover, in hypoxia, the presence of multipolar mitotic spindles and misaligned chromosomes appeared significantly evident in Ape treated cells (Figure 6A). 

### 2.3. M2 Receptors Activation Impairs GSCs Cell Survival

**Figure 7:**
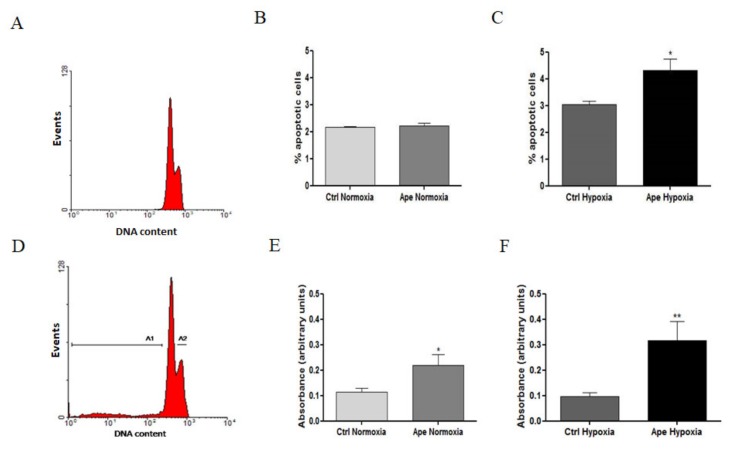
(**A**) FACS analysis of propidium iodide stained GB7 cells, showing the presence of the hypodiploid DNA content in hypoxia condition (subG1 phase; A1); (**B**) and (**C**) Percentage of apoptotic cells obtained by measuring the fraction of cells with hypodiploid DNA content in normoxia and hypoxia, and in presence or absence of 100 μM Ape (24h). (**E**) and (**F**) Levels of lactic acid dehydrogenase (LDH) activity in culture media of GB7 cells maintained in normoxia and hypoxia and in presence or absence of 100 μM Ape (24 h). The data are the mean ± SEM of the three independent experiments performed in duplicate (*t*-test;* *p* <0.05; ** *p* <0.01).

Previous studies reported the ability of Ape to negatively modulate cell survival in GBM cell lines [17,19]. In order to evaluate the effects of hypoxia and Ape on GSCs cell survival, we investigated by FACS analysis and propidium iodide staining, the fraction of cells in the sub-G1 phase both in normoxia and hypoxia and upon M2 agonist treatment. As reported in the diagram Figure 7A,D, GB7 cells treated with 100 μM Ape for 24 h showed a fraction of cells with hypodiploid DNA content and higher granularity (SSC) differently from the control sample (Figure 7A). This fraction was assumed to be cell debris derived from apoptotic events (Figure 7A,D). Figure 7B indicates that the percentage of apoptotic cells measured by FACS analysis was not significantly different between untreated and Ape-treated cells in normoxia (Figure 7B). Instead, in hypoxia, the percentage of apoptotic cells upon 24 h of Ape significantly increased (Figure 7C). 

Moreover, the LDH assay was also performed to evaluate the cell death. The results obtained indicated that the Ape treatment, both in normoxia and hypoxia, induced an increase of LDH activity in extracellular medium (Figure 7E,F). However, in the hypoxic condition the percentage of LDH activity was higher compared with normoxic cells treated with Ape (Figure 7F). 

The analysis of pro-caspase 3 by western blot analysis did not show any significant variation both in normoxia and hypoxia and after M2 agonist treatment (see Supplementary Figure S2)

## 3. Discussion

Histological evaluation of GBM tumors reveals the presence of heterogeneous cell populations with anaplastic glial cells and undifferentiated cells. The mitotic activity and cell density increase is markedly pronounced in richly vascularized niches. However, the defining feature of grade IV GBM is the presence of hypoxic necrotic foci accompanied by highly vascular stroma [24]. Hypoxia has been reported to increase the expression of the stem cell marker CD133 [12], suggesting that O_2_ deprivation increases the stability of cancer stem cells, the subpopulation involved in tumors progression and invasion. In fact, hypoxia has been shown to be correlated with enhanced tumor cell invasion and an aggressive tumor behavior [8]. Intratumoral heterogeneity in oxygenation in combination with gene mutations and epigenetic variations play a critical role in the drug resistance and in tumor recurrence [9]. Moreover, hypoxia represents an obstacle for radiotherapy; in fact, under normoxic conditions, ionizing radiation produces DNA damage, exploiting H_2_O ionization and producing free radicals that can attach to the DNA. In the absence of O_2_, DNA alterations produced by free radicals may be reduced and DNA restoring can be easier [25]. 

Although hypoxia is generally detrimental for normal cells, cancer cells have undergone extensive genetic and adaptive changes that allow them to survive in a hypoxic microenvironment.

For all these reasons, counteracting adaptive changes to hypoxia in GSCs and in general in all solid tumors, may represent a new relevant strategy for cancer therapy [11]. 

Our previous data demonstrated the ability of M2 muscarinic receptors to inhibit cell proliferation and reduce cell survival in GBM cell lines and in GSCs [16,17,18,26]. Considering the role played by hypoxia in GSC resistance, in the present work we investigated the ability of M2 agonist to impair the adaptive responses to GSC to the hypoxic conditions. 

The hypoxic environment was obtained culturing the GB7 cells in 0% O_2_, 95% N_2_, 5% CO_2_.

After only 7 h of exposure to hypoxia the Glut-1 and mir-210 appeared already significantly upregulated, but APE was able to upregulate only miR-210 expression (Supplementary Figure S1). However, any of the other markers analyzed (i.e., HIF-1α, VEGFR, PGRN, CD133) was significantly modulated at early phase of hypoxia (Supplementary Figure S1). Instead, after 24 h in hypoxia, GB7 cells increase the expression of GLUT-1 transcript and HIF-1α protein and show a more dispersed distribution compared with cells maintained in normoxia. Moreover, as expected, hypoxia induced an increase of cell number accompanied by an increase in stemness markers CD133 and PGRN, confirming that oxygen deprivation upregulates stem cell properties and cell survival also in GSCs. Interestingly, after M2 agonist treatment, a consistent decrease on cell number was observed, along with the downregulation of CD133 and PGRN transcript levels. Moreover, albeit that the expression of VEGFR, a main regulator of neo angiogenesis, was not modified in GB7 cells comparing the hypoxia and normoxia conditions, after M2 agonist treatment the expression of VEGFR transcript levels appeared significantly reduced. This suggest that M2 receptor stimulation may significantly impair the ability of tumor cells to modulate the formation of new vessels or counteracting the vasculogenic mimicry, a property of GSCs to differentiate in endothelium cells to form new tumoral blood vessels [27], contributing to increase the O_2_ levels in deprived microenvironment.

FACS analysis, performed to evaluate changes in the cell cycle progression, showed that hypoxic stress and treatment with M2 agonist induces a significant reduction of cells in active synthesis of DNA. Following exposure to the hypoxic condition, there is an enrichment of cells in the G1 phase. 

When the cells are maintained in hypoxic conditions, the M2 agonist treatment decreases the percentage of cells in S phase with as a consequent an increase of cells in the G2/M phase. However, the increased percentage of pH3-positive cells has suggested that the M2 agonist treatment in hypoxia caused a cells accumulation in M phase. Immunocytochemistry analysis for tubulin and the nuclear staining with DAPI indicated that GSCs maintained in hypoxia and treated with M2 agonist underwent to a significant increase of abnormal mitosis, with cells presenting misaligned chromosomes in metaphase plate accompanied by frequent multipolar mitotic spindles. Interestingly, M2 agonist treatment also caused an increased percentage of dead cells; in fact, apoptotic cell death was significantly increased upon M2 agonist, particularly in the hypoxic condition. The increase of LDH activity in particular in hypoxia may suggest that secondary necrotic cell death may occur as consequence of apoptotic events. This result may be a natural consequence of the increased percentage of abnormal mitosis, suggesting that the cells not able to divide correctly, undergo to cell death. The absence of significant variations of pro-caspase 3 protein after Ape treatment both in normoxia and hypoxia did not allow at least to clarify whether the apoptosis-Ape induced was caspase 3 dependent or independent. 

**Figure 8:**
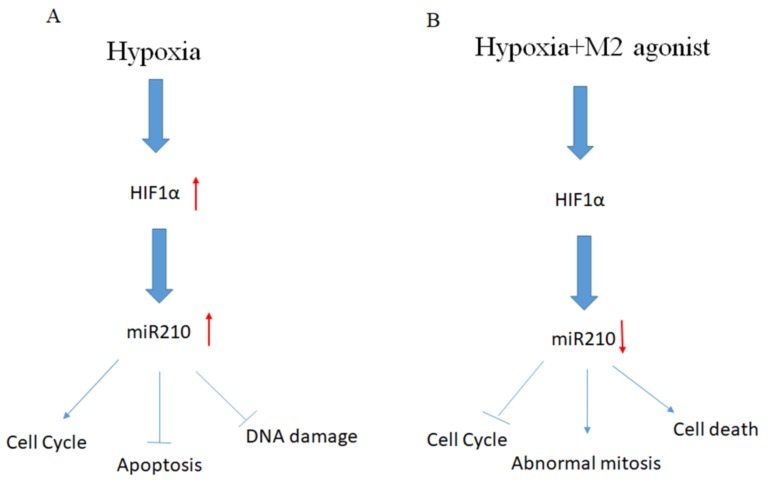
Schematic representation of the mechanisms activated in hypoxia in GSC maintained in absence (**A**) or in presence of M2 agonist Ape (**B**). Hypoxia upregulating HIF1α and miR210, promote cell survival, cell cycle progression and protects the GCSs from apoptosis and DNA damage (**A**). The treatment of GSCs with orthosteric agonist for M2 muscarinic receptor, de-regulates the adaptative responses of the cells to the hypoxic stress, causing the downregulated expression of miR210 and (PGRN), the arrest of the cell cycle progression, the increase of abnormal mitosis and decreased cell survival (**B**).

The altered cell division observed in our experimental conditions may be explained with the significant decreased expression of miR-210 levels. miR-210 is called the “master hypoxamir”. It is steadily upregulated following exposure to the hypoxic condition and it plays a central role in the adaptive changes induced by hypoxic stress [22]. One of the targets of miR-210 is the transcription factor E2F3. It promotes the regular progression of the cell cycle from phase G1 to phase S. By targeting this transcription factor, miR210 downregulation may contribute, together with other signal transduction pathways induced by the hypoxic condition, to determine an enrichment of the cell population in G1 phase [22]. This is in accordance with the ability of Ape to downregulate the miR-210 expression and to cause an increase of cells accumulated in G1 phase in normoxia conditions. However, in hypoxia, M2 agonist caused a progressive accumulation in M phase, causing aberrant mitosis. This result is in accordance with previous results obtained in GBM cells demonstrating the ability of Ape to induce genotoxic and cytotoxic effects that could well correlate with DNA damage and altered cells ability to divide correctly [19]. This aspect may be significantly increased in GB7 cells by the hypoxic condition and by the downregulation of hypoxic stress regulatory factors such as miR-210 and progranulin (Figure 8).

## 4. Materials and Methods

### 4.1. Cell Culture

The GBM CSCs GB7 were obtained from human biopsies [6,18,28]. The cells were cultured on laminin-coated dishes (1 μg/mL; Sigma-Aldrich, St. Louis, MO, USA) in serum free conditions in Euromed-N medium (EuroClone, Milan, Italy) supplemented with 1% streptomycin, 50 IU/mL penicillin (Sigma-Aldrich, St. Louis, MO, USA), 1% glutamine (Sigma-Aldrich, St. Louis, MO, USA), 1% N2 supplement (Invitrogen, Monza, Italy), 2% B27 (Invitrogen, Monza, Italy), 20 ng/mL EGF (Recombinant Human Epidermal growth factor, Peprotec, London, UK) and 20 ng/mL FGF (Recombinant Human FGF-basic, Preprotech, London, UK). The cultures were maintained at 37 °C in a humidified incubator in an atmosphere of 5% CO_2_/95% air or in hypoxia condition (0% O_2_, 95% N_2_, 5% CO_2_) in presence of a complete medium preconditioned in hypoxia for 24 h before to be added to the cells. 

### 4.2. Cell Treatments 

For reproducing the anoxic environment, we used a hypoxic/anaerobic chamber (BBLTM GasPakTM, Franklin Lakes, NJ, USA). The system was set up at 37 °C in 5% CO_2_, 95% N_2_. Cells were transferred into the humidified chamber and incubated with the appropriate media for 24 h. The cells were then detached and analyzed according to the experimental plan. Control cells were incubated under normoxic conditions. 

Arecaidine Propargyl Ester hydrobromide (Ape, Sigma-Aldrich, Milan, Italy) is a synthetic alkaloid obtained from modification of areca nut arecaidine. Its ability to selectively bind M2 muscarinic subtype has been largely demonstrated by pharmacological binding and M2 knockdown experiments [17,18]. Cells were treated with 100 µM Ape, considering that this concentration was able to negatively control cell growth both in GBM cell lines and in GSCs, as previously demonstrated [18]. 

### 4.3. Total RNA Extraction 

Total RNA was extracted from cells using the Invisorb Spin Cell RNA kit (Invitek), according to the manufacturer’s instruction [20]. RNA concentration and purity were detected using the NanoDrop Lite Spectrophotometer (Thermo, Dreieich, Germany). 

### 4.4. Retro-Transcription and Real Time PCR

RNA samples (2 μg) was reverse transcribed for 60 min at 37 °C with Random Primers (Promega, Madison, WI, USA) and M-MLV reverse transcriptase (Promega, Madison, WI, USA). 

The expression of the transcripts was evaluated by quantitative RT-PCR analysis using the specific primers or TaqMan Probes (Table 1). 

A quantity of 50 ng of each cDNA, was used as template in each tube for real time-PCR assay. Real-time RT-PCR was performed with SYBR Green Mastermix (Promega, Mi, Italy) and primers (final concentration 300 nM) added at the respective reaction tubes and analyzed by Rotor–Gene Q-Pure detection (Qiagen, Hilden, Germany). All samples were run in triplicate. The real time-PCR conditions included a denaturing step at 95 °C for 3 min followed by 40 cycles at 95 °C for 30 s, 60 °C for 30 sec and 75 °C for 45 s. Two cycles were included as final steps: one at 95 °C (1 min) and the other at the annealing temperature specific for each couple of primers used (1 min). Data were normalized with GAPDH housekeeping gene and the ΔCt method was used to determine the fold changes in the gene expression. 

The analysis of expression for human PGRN and GLUT-1 was performed by using a specific TaqMan probes (ThermoFisher Scientific, Milan, Italy). 

### 4.5. miRNA Expression

For miR-210 analysis, cDNA was obtained by reverse transcription with miRCURY LNA^TM^ Universal RT microRNA PCR (Exiqon-Qiagen, Hilden, Germany). Final reaction volumes were 10 µL containing 2 µL of reaction buffer, 1 μL Reverse transcription mix, 5 µL of RNase-free water and 2 µL of template RNA. Real-time miRNA detection was performed using miRCURY LNA^TM^ Universal RT microRNA PCR ExiLENT SYBR^®^ Green (Exiqon-Qiagen, Hilden, Germany) with 10 µL mixtures containing 5 µL of SYBR Green PCR Master mix, 1 of Primer Assay, and 4 µL of cDNA template. To normalize the miRNA expression, U6 snRNA (small nuclear RNA) expression was also quantified. The parameters for PCR amplification were 95 °C for 10 min followed by 40 cycles of 95 °C for 10 s and 60 °C for 1 min. miRNA qPCR was performed on ABI 7500 instrument (Applied Biosystems, Carlsbad, CA, USA). Each reaction was run in triplicate with a non-template control. The relative expression was calculated by using the comparative delta Ct method. Data were expressed as fold-change relative to the mean of U6 values.

### 4.6. Western Blot Analysis

Cells were harvested in RIPA buffer (25 mM Tris–HCl pH 7.4, 150 mM NaCl, 1.0% NP-40, 0.1% SDS, 1% Na-deoxycholate), containing a protease inhibitor cocktail (Sigma-Aldrich, St. Louis, MO, USA), 1 mM Na_3_VO_4_ and 5 mM NaF, for 1 h in ice. The cellular extracts were solubilized in 4× Laemmli sample buffer and boiled for 5 min. The protein extracts were run on SDS-polyacrilamide gel (SDS-PAGE) and transferred to Polyvinylidene Difluoride (PVDF) sheets (Merck Millipore, Darmstadt, Germany). Membranes were blocked for 30′ in 5% of non-fat milk powder (Sigma-Aldrich, St. Louis, MO, USA) in Tris-buffered saline (TBS) containing 0.1% Tween-20, and then incubated with the antibodies overnight at 4 °C. The blots were then washed three times with TBS/Tween-20, then incubated with anti-HIF-1α (HIF-1-α 1:200; Santa Cruz Biotechnology, Texas, USA) or anti-pro-caspase 3 (1: 1000, Santa Cruz Biotechnology, Texas, USA) primary antibodies, washed again for 3 times with TBS/Tween-20 and incubated with secondary antibodies conjugated to horseradish-peroxidase. Beta-actin (1:800; Immunological Sciences, Rome, Italy) was used as reference protein. The reaction was revealed by ECL chemiluminescence reagent (Immunological Science, Rome, Italy). The bands were detected by exposition to Chemidoc (Molecular Imager ChemiDoc XRS + System with Image Lab Software, Biorad, CA, USA) and band intensities were quantified by optical density (ImageJ software, National Institutes of Health, Bethesda, Maryland, USA). 

### 4.7. Flow Cytometry Analysis

The cells were plated onto flask t25 at density of 1 × 10^6^ cells/flask. The day after plating, the cells were treated with 100 µM of preferential M2 receptor agonist Ape (Sigma-Aldrich, St. Louis, MO, USA) and kept at 37 °C either in normoxia or in hypoxia condition for 24 h. Then, the cells were incubated for 90 min with 45 μM bromodeoxyuridine (final concentration) (BrdUrd, Sigma-Aldrich, St. Louis, MO, USA), collected by trypsinization, centrifuged for 3 min at 1500 rpm, washed with Phosphate Buffered Saline (PBS) three times and then fixed in methanol/PBS 1:1 (*v*/*v*). 

To identify cells in S phase, DNA content and BrdUrd incorporation were determined by staining with propidium iodide (PI) and anti-BrdU antibody, respectively. By incubating the cells in 3N HCl for 45 min at room temperature, DNA was denaturated, followed by neutralization with 0.1 M sodium tetraborate. Samples were then incubated with monoclonal anti-BrdUrd antibody (1:50 *v*/*v*; Dako, MI, Italy) for 1 h at room temperature, washed twice with 0.5% Tween-20 in PBS and incubated for 30 min with anti-mouse Alexa fluor 488-conjugated antibody (dil 1:1200; Invitrogen, Monza, Italy). Samples were washed twice with PBS and stained with 10 μg/mL PI for 15 min at RT. 

The percentage of cells accumulated in M phase was evaluated by the staining with phospho-H3 (ser-10). Cells was plated at density of 1 × 10^6^ cells/flask. The day after plating, the cells were treated with 100 µM Ape (Sigma-Aldrich, St. Louis, MO, USA) and kept at 37 °C either in normoxia or in hypoxia condition for 24h or treated with Nocodazole (0.2μg /μL), used as positive control. Samples were then incubated with monoclonal anti-phospho H3 (Ser10) (dilution 1:300, Millipore, Mi, Italy) for 60 min at RT, washed with 0.5% Tween-20 in PBS and incubated for 30 min with anti-mouse Alexa fluor 488-conjugated antibody (dilution 1:1200, Invitrogen, Monza, Italy). Finally, the samples were stained with 10 μg/mL propidium iodide (PI). 

Flow cytometry analysis was performed with a flow cytometer Coulter Epics XL with 488 nm wavelength excitation and 10^4^ events were collected for each sample. Monoparametric (DNA histograms) and biparametric (BrdUrd incorporation *vs* DNA content) analysis were performed using WinMDI 2.9 software (Scripps Research Institute, La Jolla, CA, USA).

### 4.8. Apoptotic Cell Detection

Cell dead was evaluated by trypan blue staining. Apoptotic cells were evaluated by flow cytometry analysis by propidium iodide (PI) staining. The cells were plated at density of 1 × 10^6^ cells/flask and the day after treated with 100 µM APE (Sigma-Aldrich, St. Louis, MO, USA) for 24 h either in normoxia or hypoxia. Then the cells were collected and suspended in 2 mL of PBS buffer containing 0.1% Triton X-100 (Sigma-Aldrich, St. Louis, MO, USA) and incubated for 5 min at RT. Cells were subsequently stained with 10 μg/mL PI and analyzed by using a Coulter Epics XL flow cytometer. For each sample, 10^4^ events were recorded. Cells with a hypodiploid DNA content and a higher granulosity (SSC) at G0-G1phase (sub G1) were quantified as apoptotic cells [29,30]. Analysis of pro-caspase 3 protein was evaluated by western blot analysis (see western blot section).

### 4.9. Immunocytochemistry

GB7 cells were plated onto 35-mm diameter dishes in complete medium, treated with 100 µM APE (Sigma-Aldrich, St. Louis, MO, USA) and kept at 37 °C either in a normoxia or in hypoxia condition for 24h. Then, the cells were washed three times with PBS, fixed with 4% paraformaldehyde for 20 min at RT, washed three times in PBS and permeabilized by treatment with PBS containing 0.1% Triton X-100, 10% NGS for 30 min at RT. The cells were then incubated overnight at 4 °C with anti-α-tubulin antibody (dilution 1:100; Sigma Aldrich, MI, Italy) in PBS containing 0.1% Triton X-100 1% NGS. The next day, the cells were washed twice in PBS and incubated for 1 h at RT with an Alexa 594 conjugated goat anti-mouse (IgG diluted 1:2000 in PBS + 0.1% Triton X-100 + 1% NGS, Promega, Madison, WI, USA) and washed three times with PBS. The cells were finally mounted with 30 μL of Anti Fade Mounting Medium with DAPI (Immunological Science, Rome, Italy). Negative controls were obtained by omitting the primary antibody. For the evaluation of abnormal mitosis, the ratio between total abnormal methaphases/mitotic cells was calculated. Ten photographic fields for each sample were considered. Each sample was produced in triplicate. 

### 4.10. LDH Assay 

Cell death was quantified by a lactic acid dehydrogenase (LDH) release assay. LDH activity was assessed by determining the amount of NADH generated in a reaction between NAD(+) and lactate. LDH activity of each supernatant was determined at 490 nm with an LDH Cytotoxicity Detection Kit (Takara, Japan) and a microplate reader (Sunrise, Tecan, Swiss). Results were normalized to the background levels of LDH.

### 4.11. Statistical Analysis

Student’s t test and one-way ANOVA test followed by Bonferroni’s post test were used to evaluate statistical significance within the different samples. The results were considered statistically significant at *p* < 0.05 (*), *p* < 0.01 (**) and *p* < 0.001 (***).

## 5. Conclusions

The results obtained demonstrate the ability of M2 agonist Ape to counteract the adaptive responses to hypoxia conditions in GSCs, making them less resistance to the hypoxic stress. 

The downregulated expression, upon Ape treatment, of factors typically overexpressed in hypoxic conditions, such as miR-210, progranulin and VEGFR, suggest that the M2 receptor activation may be a strategic therapeutic tool to counteract GSCs cell proliferation, survival and stemness properties that normally promote tumor aggressiveness typically associated with O_2_ deprivation. 

## Figures and Tables

**Table 1:** Sequences of the primers used in qRT-PCR.

	Forward	Reverse
**18S**	5′-CCAGTAAGTGCGGGTCATAAGC -3′	5′-AACGATCCAATCGGTAGTAGCG -3′
**CD133**	5′-GCATTGGCATCTTCTATGGTT-3′	5′-CGCCTTGTCCTTGGTAGTGT-3′
**VEGFR**	5′-GTGACCAACATGGAGTCGTG-3′	5′-TGCTTCACAGAAGACCATGC-3′
**HIF-1α**	5′-TGATGACCAGCAACTTGAGG-3′	5′-TTGATTGAGTGCAGGGTCAG-3′

## Supplementary Materials

Supplementary materials can be found at https://www.mdpi.com/1422-0067/21/5/1700/s1.
