# Supplementary material for: M2 Receptor Activation Counteracts the Glioblastoma Cancer Stem Cell Response to Hypoxia Condition
Source: Int J Mol Sci. 2020 Mar 2;21(5):1700. doi: 10.3390/ijms21051700 (PMC7084794; doi:10.3390/ijms21051700)
Supplement: Supplementary file 1 [file ijms-21-01700-s001.pdf]

International Journal of
Molecular Sciences

MDPI

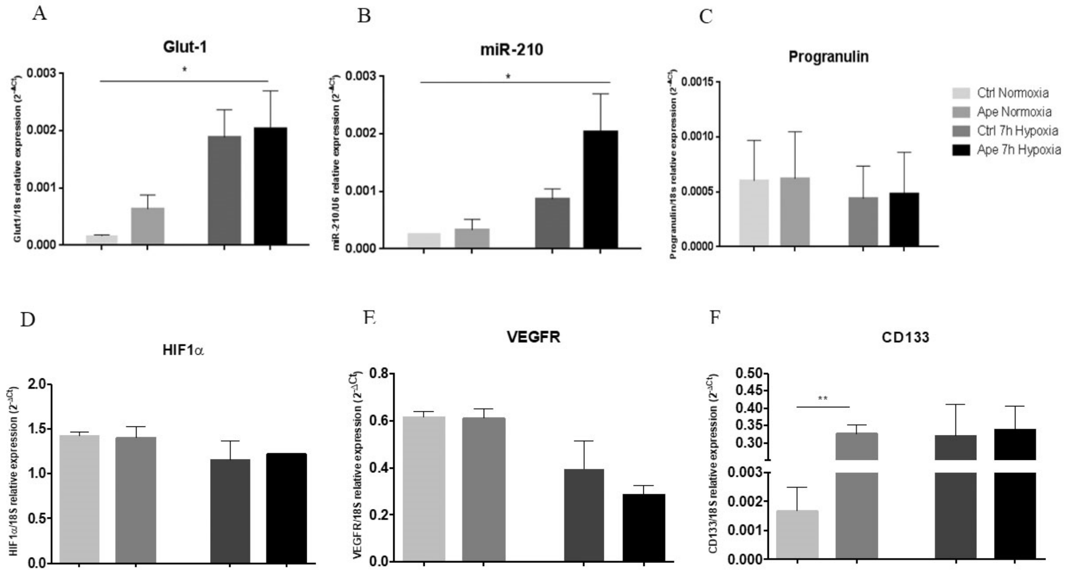

Supplementary Fig. S1. qRT-PCR analysis for Glut-1 (**A**), miR-210 (**B**), Progranulin (**C**), HIF1α (**D**), VEGFR (**E**) and CD133 (**F**) expression following 7h of hypoxia, in absence and presence of 100 μM Ape. The data are the mean±SEM of the three independent experiments performed in duplicate (ANOVA test, * p<0.05; t-test, ** p<0.01).

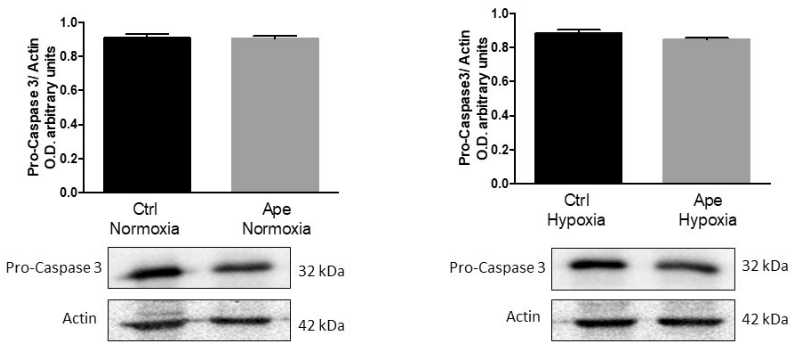

Supplementary Fig. S2. Western blotting analysis for pro-caspase 3 expression in GB7 cells in normoxia and 48h hypoxia, in absence or presence of 100 μM Ape (48h). The graph shows the densitometric analysis of the bands obtained after normalization with the β-actin as reference protein. The data are the mean±SEM of the three independent experiments.
